# Retraction: Zhang, K., et al. Knockdown of Snail Sensitizes Pancreatic Cancer Cells to Chemotherapeutic Agents and Irradiation. *Int. J. Mol. Sci.* 2010, *11*, 4891–4904, doi:10.3390/ijms11124891 and Zhang, K., et al. RNA Interference Targeting Slug Increases Cholangiocarcinoma Cell Sensitivity to Cisplatin via Upregulating PUMA. *Int. J. Mol. Sci.* 2011, *12*, 385–400, doi:10.3390/ijms12010385

**DOI:** 10.3390/ijms18071483

**Published:** 2017-07-10

**Authors:** International Journal of Molecular Sciences Editorial office

**Affiliations:** MDPI AG, St. Alban-Anlage 66, 4052 Basel, Switzerland

The two articles [1,2] published in the *International Journal of Molecular Sciences* will be retracted. An investigation of the peer review process has concluded that it was compromised via the use of fake reviewer email addresses provided by the authors. MDPI takes the integrity of its review process very seriously. Since bringing the management of the peer review process in-house in 2006 [3], we have had in place criteria for inviting peer reviewers which include confirmation of their identity, especially for reviewers suggested by authors. In the years since the publication of [1] and [2], we have further updated the technology available to editors in order to mitigate against the possibility that review reports could be faked by the authors.

An internal investigation has concluded that for these two papers the procedures were not correctly followed, for which we apologize to the readers of the journal. The investigation covered all MDPI journals and these were the only problematic cases discovered.

We believe that the vast majority of authors submit manuscripts in good will, however we remain vigilant for suspicious activity and strive to have robust procedures in place to ensure that such cases can be discovered prior to publication.
